# Intravenous immunoglobulin immunotherapy for coronavirus disease‐19 (COVID‐19)

**DOI:** 10.1002/cti2.1198

**Published:** 2020-10-16

**Authors:** Caroline Galeotti, Srini V Kaveri, Jagadeesh Bayry

**Affiliations:** ^1^ Service de Rhumatologie Pédiatrique Centre de Référence des Maladies Auto‐Inflammatoires Rares et des Amyloses CHU de Bicêtre le Kremlin Bicêtre France; ^2^ Institut National de la Santé et de la Recherche Médicale Centre de Recherche des Cordeliers Sorbonne Université Université de Paris Paris France

## Abstract

Intravenous immunoglobulin (IVIG), a pooled normal IgG from several thousand healthy donors and one of the commonly used immunotherapeutic molecules for the management of autoimmune and inflammatory diseases, has been explored for the treatment of coronavirus disease‐19 (COVID‐19). Although placebo‐controlled, double‐blind randomised clinical trials are lacking, current data from either retrospective, case series or open‐label randomised controlled trials provide an indicator that IVIG immunotherapy could benefit severe and critically ill COVID‐19 patients. See alsoShao et al.

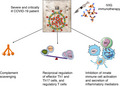

The coronavirus disease‐19 (COVID‐19) pandemic caused by the novel severe acute respiratory syndrome coronavirus 2 (SARS‐CoV‐2) has caused widespread morbidity and mortality across the globe. Most of the affected patients depend on mechanical ventilation. The SARS‐CoV‐2 infection triggers massive influx of activated immune cells to the lungs causing cytokine storm and severe lung lesions.^1^ Although our understanding of the pathogenesis of the disease and patient management have drastically improved recently, no specific pharmacological treatment is available yet. As cytokine storm is the major cause of morbidity and mortality, several drugs such as steroids that target inflammation, inhibitors of signalling molecules and also immunotherapies such as neutralising antibodies to IL‐6 and GM‐CSF; recombinant IL‐1 receptor antagonist, blocking antibodies to IL‐6 receptor and intravenous immunoglobulin (IVIG) have provided encouraging results.^2^ While the aforementioned immunotherapies target a specific molecule, IVIG exerts its therapeutic benefits by several mutually nonexclusive mechanisms targeting diverse arms of the inflammatory immune response.

## Intravenous immunoglobulin

Intravenous immunoglobulin, a therapeutic normal IgG isolated from the pooled plasma of several thousand healthy donors, is one of the frequently used immunotherapeutic molecules for treating various autoimmune and inflammatory diseases.^3^ In general, the dose of IVIG for the therapeutic purposes is 2 g kg^–1^ infused either one, two or five consecutive days. In addition to clinically approved pathological conditions including Kawasaki disease, immune thrombocytopenic purpura, inflammatory myopathies, Guillain–Barré syndrome, graft‐vs‐host disease and blistering diseases, IVIG has been explored in over 100 diseases as an off‐label drug. IVIG exerts its therapeutic benefits in autoimmune diseases by several mutually nonexclusive mechanisms targeting both soluble and cellular mediators of the inflammatory immune response. These mechanisms include complement scavenging, neutralisation of autoantibodies by idiotypic network; enhancement of degradation of autoantibodies by neonatal Fc receptor saturation; inhibition of activation of various innate immune cells including dendritic cells, monocytes, macrophages and neutrophils, and secretion of inflammatory mediators; suppression of effector T helper cells Th1 and Th17, and reciprocal enhancement of immunoprotective regulatory T cells (Tregs); and blockade of B‐cell activation.^4^ These multitude anti‐inflammatory mechanisms and proven safety record of the drug prompted clinical evaluation of IVIG in the management of severe and critically ill COVID‐19 patients.

## IVIG immunotherapy for the treatment of Covid‐19

In a recent article published in *Clinical & Translational Immunology*, Shao *et al*.^5^ presented a multicentre retrospective cohort study evaluating the efficacy of IVIG in 325 severe and critically ill COVID‐19 patients admitted to hospitals in southern China between December 2019 and March 2020. In their cohort, 174 patients (64% male, median age 61 years) received IVIG, while 151 patients (51% male, median age 56 years) did not receive this therapy. Patients received IVIG at a dose of 0.1–0.5 g kg^−1^ day^−1^ for the duration of 5–15 days. Additional therapies include antibiotics, anti‐viral drugs and steroids as per guidelines of China. Their analyses showed that early administration (≤ 7 days postadmission) of high‐dose (> 15 g day^−1^) IVIG improves the prognosis of critical‐type patients with COVID‐19.

This multicentre study has certain limitations. First, it is a retrospective study. Second, a wide range of IVIG dose and varying duration of treatment were employed. Third, there is a lack of analyses of various inflammatory cytokines and immune cells. Randomised, placebo‐controlled clinical studies with mechanistic insight are critical in assessing the full spectrum of IVIG efficacy and window of treatment in COVID‐19. As anti‐inflammatory effects of IVIG normally rely on a high dose (2 g kg^–1^), this dosage should be considered for future clinical trials.

Currently, data are available from seven reports including the multicentre study of Shao *et al*. that explored IVIG immunotherapy in the management of COVID‐19 (Table 1).^5^, ^6^, ^7^, ^8^, ^9^, ^10^, ^11^ These studies are mostly case series or retrospective, with an exception of one study that was randomised open‐label trial. Although retrospective, data from Shao *et al*. included large number of COVID‐19 patients compared to other studies, and hence, data are more reliable.^5^ Importantly, a retrospective study of Xie *et al*.^9^ also confirmed the therapeutic benefits of IVIG if therapy is initiated early. Another report documented that short‐term moderate‐dose corticosteroid plus IVIG (20 g day^−1^) might effectively benefit COVID‐19 patients who did not respond to low‐dose therapy (10 g day^−1^).^6^ The only randomised controlled study on IVIG also combined methylprednisolone.^8^ In view of the corticosteroid combination, it is difficult to evaluate the therapeutic benefits of IVIG in these trials. Another study combined IVIG with anakinra, a recombinant modified IL‐1 receptor antagonist.^11^ Thus, in view of associated therapies, the therapeutic benefits of IVIG have to be evaluated carefully. It is important to note that studies in autoimmune and inflammatory diseases have reported better outcomes if IVIG is combined with steroids. Also, IVIG has steroid‐sparing effects.

**Table 1:** Overview of clinical studies on the IVIG immunotherapy in COVID‐19 patients^a^

Study	Location/country	Nature of the study	No. of patients	Age (years)	Gender	IVIG dose	Duration of therapy	Additional therapies	Clinical outcome of IVIG therapy	Laboratory findings
Shao *et al*.^5^	Wuhan, Guangzhou, Shenzhen (China)	Multicentre retrospective	IVIG: 174 Non‐IVIG: 151	IVIG: 61^b^ (50–69) Non‐IVIG 56^b^ (38–67)	IVIG – male: 112 (64%) Non‐IVIG – male: 77 (51%)	0.1–0.5 g kg^−1^ day^−1^	5–15 days	Corticosteroids antivirals, antibiotics	Improved 28‐day mortality Improved organ function in critically ill patients Reduced inflammatory response in critically ill patients Reduced 60‐day mortality in critically ill patients who received early (≤ 7 days postadmission) and high‐dose (≥ 15 g day^−1^) IVIG therapy	↓ IL‐6 ↓ CRP
Zhou *et al*.^6^	Hunan (China)	Single‐centre retrospective	10	51.60 ± 15.46^c^ (29–68)	Male: 8 (80%)	10 g day^−1^ followed by 20 g day^−1^	17–24 days	Corticosteroids, lopinavir, ritonavir, interferon, antibiotics	Improved oxygenation index Improved pulmonary lesions (70%) Reversal of disease progression (90%)	↓ CRP ↓ CK ↑ lymphocytes
Cao *et al*.^7^	Wuhan (China)	Case series	3	41.67 ± 12.42^c^ (34–56)	Male: 2 (67%)	25 g day^−1^	5 days	Methylprednisolone Moxifloxacin	Improved clinical status Resolution of pulmonary lesions Return of oxygen saturation and discontinued oxygen supplement	↓ CRP ↑ Platelets
Sakoulas *et al*.^8^	San Diego, La Mesa (USA)	Open‐label randomised controlled trial	IVIG: 16 Non‐IVIG (standard of care): 17	IVIG: 58^b^ Non‐IVIG: 51	IVIG – male: 10 (63%) Non‐IVIG – Male: 10 (59%)	0.5 g kg^−1^ day^−1^	3 days	Methylprednisolone, remdesivir, convalescent plasma	Reduced ICU days Reduced ventilator patient days	↓ Ferritin ↓ IL‐6
Xie *et al*.^9^	Wuhan (China)	Single‐centre retrospective	58	63^b^ (29–86)	Male: 36 (62%)	20 g day^−1^	Not precise	Corticosteroids, abidor, moxifloxacin	In patients who received IVIG therapy ≤ 48 h upon hospital admission Reduced 28‐day mortality Reduced hospital stays Reduced ICU stays Reduced mechanical ventilation	Not available/analysed
Mohtadi *et al*.^10^	Ilam (Iran)	Case series	5	60.4 ± 6.8^c^ (50–66)	Male: 1 (20%)	0.3–0.5 g kg^−1^ day^−1^	5 days	Hydrocortisone, vancomycin, meropenem, azithromycin	Improved clinical symptoms Resolution of pulmonary lesions Return of oxygen saturation	Not available/analysed
Zantah *et al*. ^11^	Philadelphia (USA)	Single‐centre retrospective	Anakinra/IVIG: 51 Tocilizumab: 33	Anakinra/IVIG: 62.7 ± 12.3^c^ Tocilizumab: 57.4 ± 14.6	Anakinra/IVIG – male: 33 (65%) Tocilizumab – male: 20 (61%)	0.5 g kg^−1^ day^−1^	3 days	Methylprednisolone	In both the groups Improved clinical outcome Reduced ICU requirement and stays Reduced hospital stays Reduced mechanical ventilation duration	↓ Ferritin ↓ LDH in living patients of both the groups

CK, creatine kinase; CRP, C‐reactive protein; ICU, intensive care unit; LDH, lactate dehydrogenase.

^a^ Studies that contain at least three patients were considered.

^b^ Median.

^c^ Mean.

## Possible mechanisms of action of IVIG in COVID‐19

**Figure 1:**
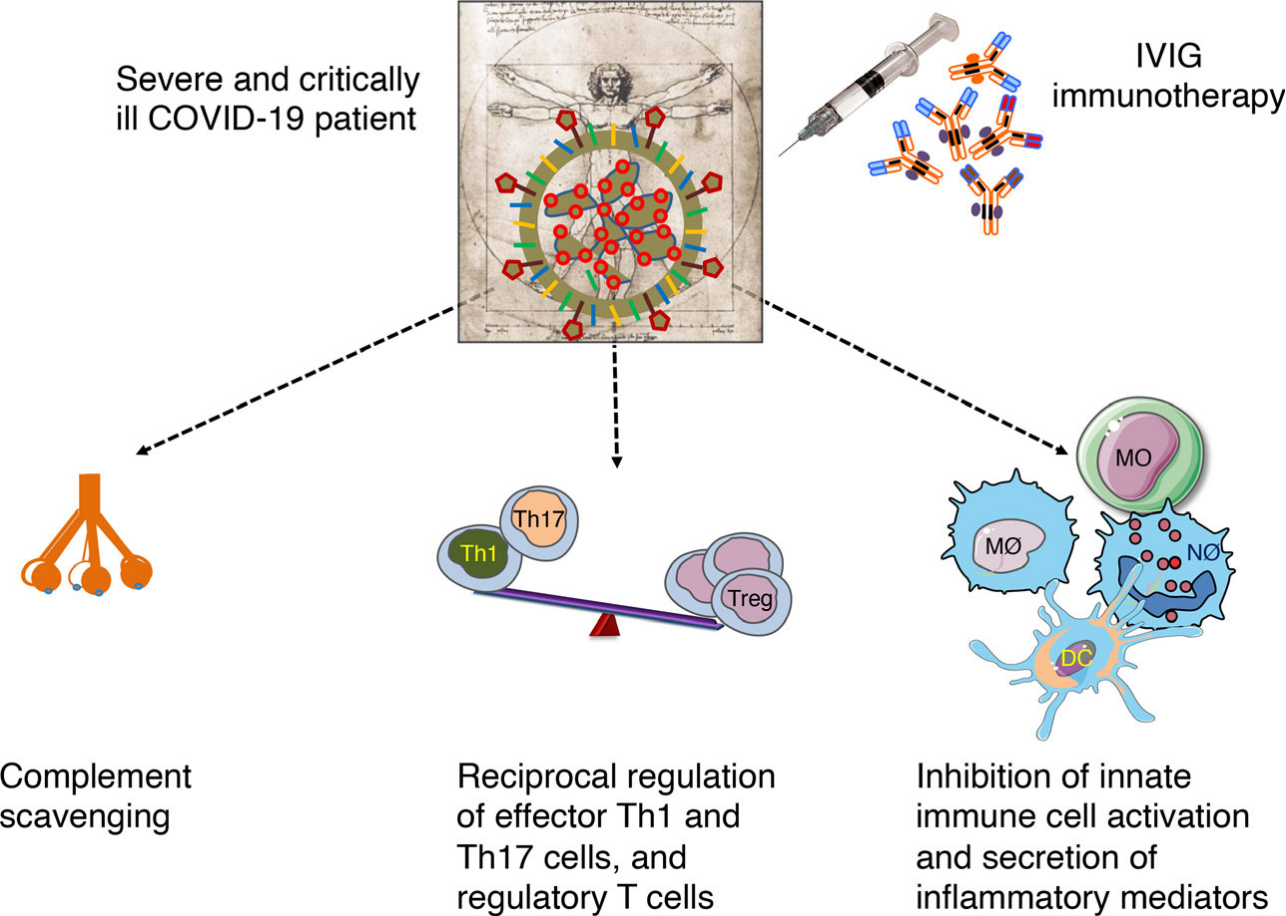
Possible mechanisms of action of intravenous immunoglobulin (IVIG) in coronavirus disease‐19 (COVID‐19). It is not yet clear how IVIG benefits severe and critically ill COVID‐19 patients. Several studies have reported that IVIG reduces IL‐6 and C‐reactive protein in the COVID‐19 patients suggesting that IVIG targets inflammatory process. Based on the known mechanisms of IVIG demonstrated in autoimmune and inflammatory diseases, and considering the pathophysiology of COVID‐19, we propose that IVIG might target cytokine storm in COVID‐19 patients by complement scavenging; reciprocal regulation of effector Th1 and Th17 cells, and regulatory T cells (Treg); and inhibition of innate immune cell activation and secretion of inflammatory mediators. DC, dendritic cell; MO, monocyte; MØ, macrophage; NØ, neutrophil.

How does IVIG benefit COVID‐19 patients? Although the mechanisms are not yet precisely understood, reduction in the inflammatory mediators following IVIG therapy (Table 1) suggests that IVIG might target cytokine storm in severe and critically ill COVID‐19 patients by complement scavenging, inhibition of innate immune cells and effector T‐cell activation, and expansion of Tregs (Figure 1). Although current batches of IVIG could neutralise seasonal coronavirus, they lack cross‐neutralising antibodies to SARS‐CoV‐2.^12^ Therefore, passive virus neutralisation appears to be not responsible for the beneficial effects of IVIG. Recently, it has been found that SARS‐CoV‐2 encodes a superantigen motif near its S1/S2 cleavage site that might trigger cytokine storm.^13^ As IVIG contains antibodies reacting against SARS‐CoV‐2 antigens,^14^ IVIG might inhibit superantigen‐mediated T‐cell activation and cytokine release.

## Biomarkers of IVIG response in COVID‐19 patients are essential

From the experience of treating the autoimmune diseases, it is clear that IVIG is not a magic bullet and, understandably, all COVID‐19 patients do not respond to IVIG therapy. The current evidence from Shao *et al*. and other studies suggests that early initiation of IVIG therapy and high‐dose infusion could benefit COVID‐19 patients. Therefore, studies are required to identify potential biomarkers of IVIG response in COVID‐19 patients. As severe and critically ill COVID‐19 patients need to receive immediate medical care, healthcare personnel should be able to analyse these predictive biomarkers by easy and rapid methods without sophisticated instruments. Shao *et al*. and Sakoulas *et al*. indicate that IVIG decreases IL‐6 levels in the plasma. Majority of the studies have also reported that C‐reactive protein (CRP) is downregulated following IVIG therapy (Table 1). Although CRP is a general marker of inflammation and is not specific for IVIG therapy, post‐IVIG levels of CRP could be investigated further as a biomarker of IVIG response. In addition, pre‐IVIG levels of inflammatory mediators such as G‐CSF, IFN‐γ, IL‐1β, and IL‐6; the levels of various innate cells (such as monocytes and neutrophils) and adaptive immune cells (such as Th1 and Th17) could also be considered to predict whether patients could benefit from IVIG therapy.^15^

## Perspectives

In addition to COVID‐19, SARS‐CoV‐2 infection is also linked to other complications such as triggering of autoimmune and inflammatory diseases including Kawasaki‐like paediatric inflammatory multisystemic syndrome, Guillain–Barré syndrome and idiopathic thrombocytopenic purpura. IVIG therapy is also beneficial in these SARS‐CoV‐2 infection‐associated rare pathological manifestations.^16^, ^17^, ^18^

Several phase 2, 3 and 4 clinical trials (NCT04500067, NCT04411667, NCT04480424, NCT04432324, NCT04350580, NCT04400058, NCT04261426, NCT04403269) including double‐blind, placebo‐controlled, randomised studies are registered in the United States, France, Spain, China and Ukraine. As clinicians are now better equipped with armory of drugs to treat COVID‐19 patients and because of the cost associated with IVIG therapy, these registered studies might not get required number of patients to complete the investigation. Also, IVIG is a life‐saving drug for primary immunodeficient patients and first‐line therapy for many autoimmune and inflammatory diseases. In view of worldwide shortage of IVIG, priority should be given to the patients whose survival is dependent on IVIG while continuing the investigation on COVID‐19. Alternatively, in view of broad‐spectrum anti‐inflammatory properties of IVIG, this therapy could be combined with IL‐6‐ and IL‐1‐targeted immunotherapies that showed favorable responses^19^, ^20^ to explore additive or synergistic therapeutic benefits.

## Author contributions

**Caroline Galeotti:** Writing‐original draft; Writing‐review & editing. **Srini V Kaveri:** Writing‐review & editing. **Jagadeesh Bayry:** Conceptualization; Writing‐original draft; Writing‐review & editing.

## Conflict of interest

The authors declare no conflict of interest.
